# Supplementary material for: An immunologically active, adipose-derived extracellular matrix biomaterial for soft tissue reconstruction: concept to clinical trial
Source: NPJ Regen Med. 2022 Jan 14;7:6. doi: 10.1038/s41536-021-00197-1 (PMC8760240; doi:10.1038/s41536-021-00197-1)
Supplement: Supplementary file 1 — Supplementary Figures and Tables [file 41536_2021_197_MOESM1_ESM.pdf]

SUPPLEMENTARY FIGURES

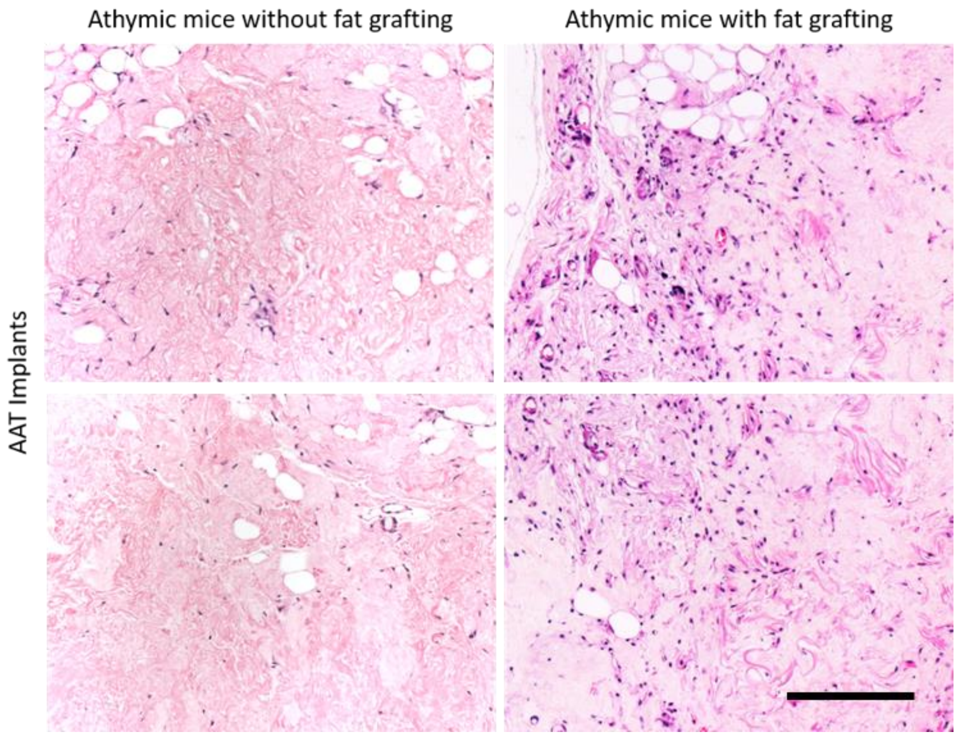

Supplementary Figure 1. Increased cellular infiltration of AAT implants is observed in athymic mice also injected distally with human lipoaspirate. Scale bar represents 100 µm.

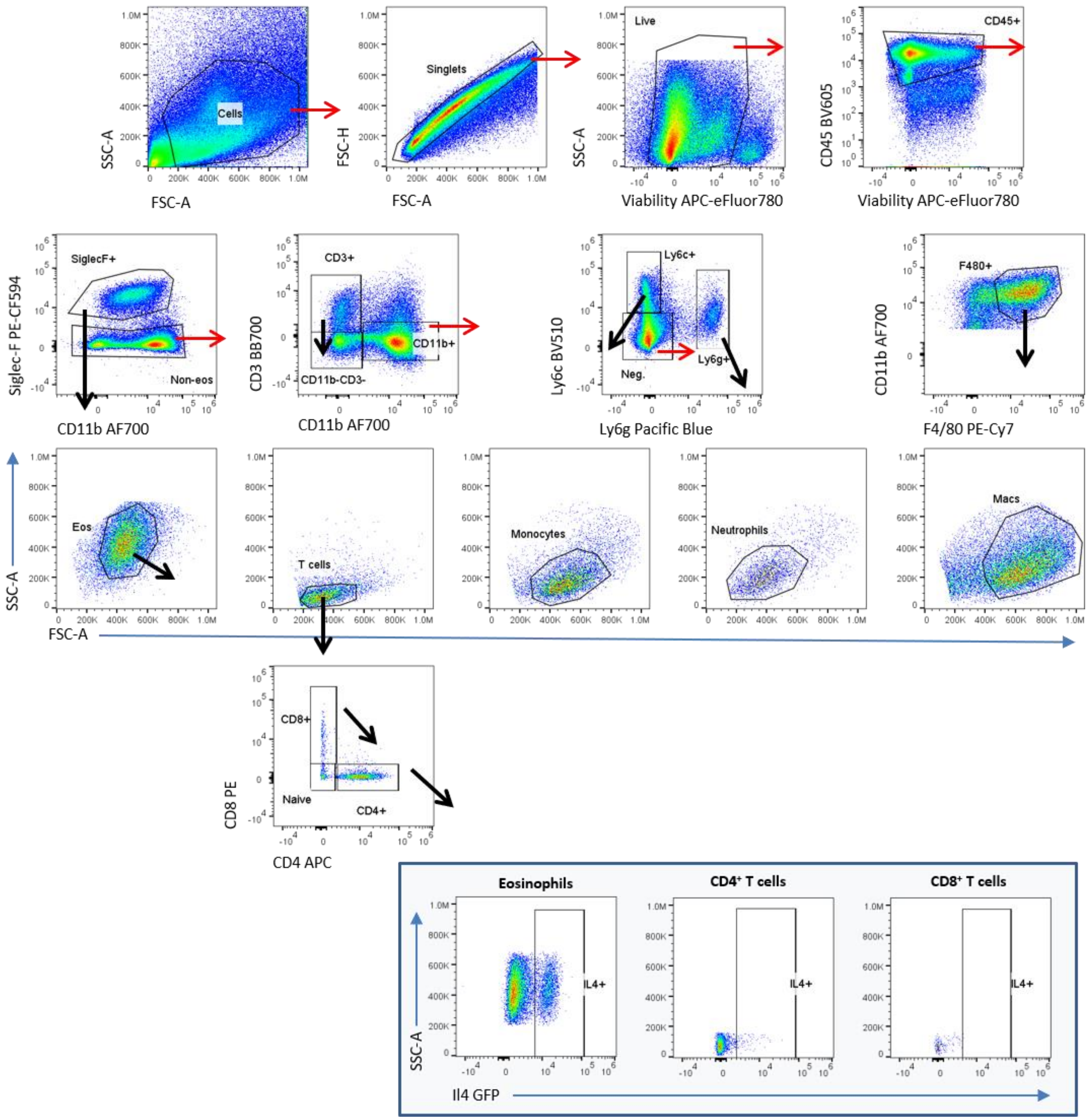

Supplementary Figure 2. Gating schema for flow cytometry experiments assessing immune populations and Il4 expression in 4get mice with VML wounds.

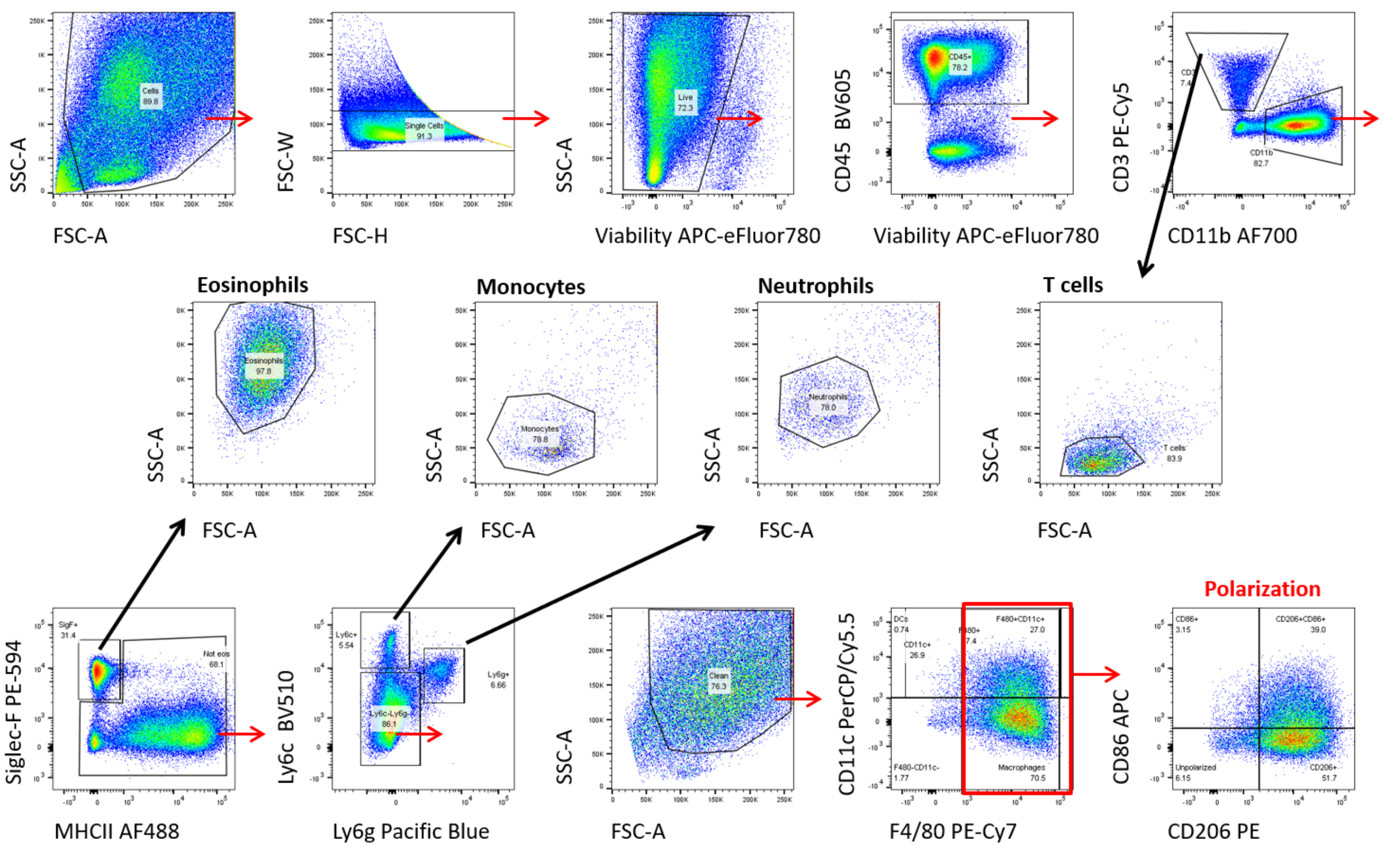

Supplementary Figure 3. Gating schema for flow cytometry experiments assessing macrophage polarization in C57BL/6 mice with VML wounds.

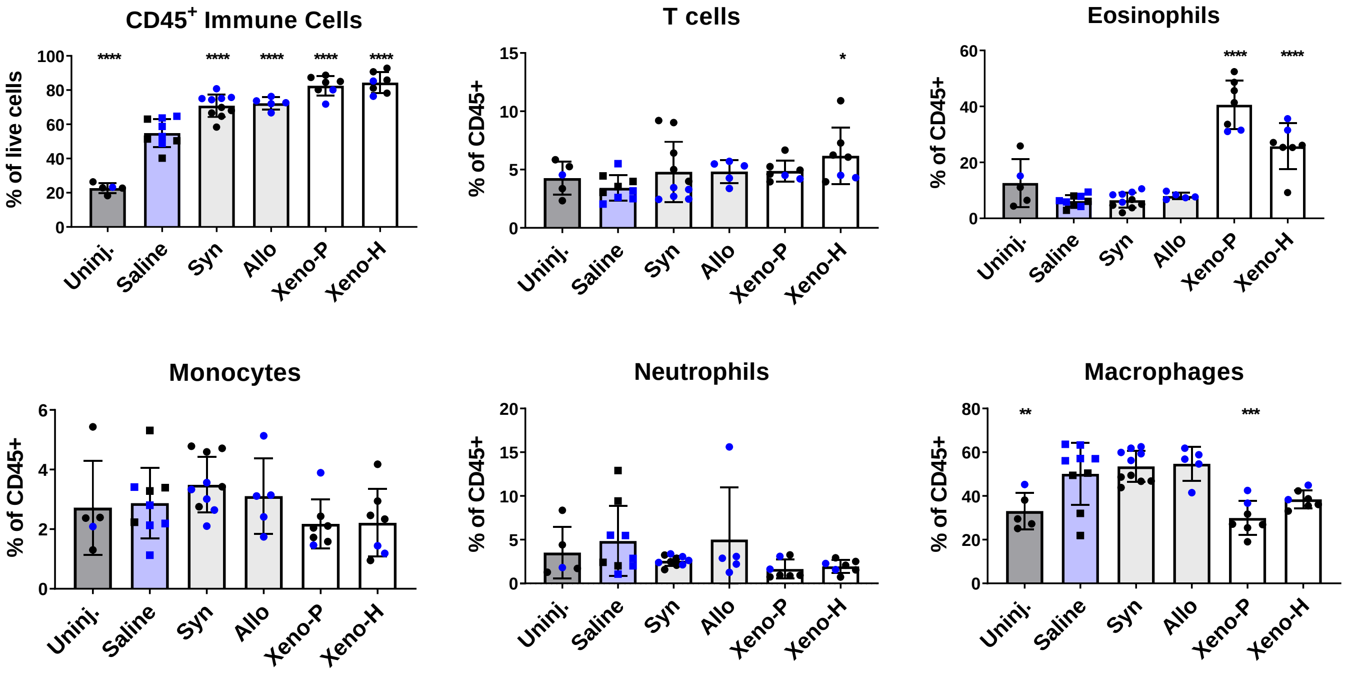

Supplementary Figure 4. Immune recruitment profile of syngeneic, allogeneic, and xenogeneic AATs in C57BL/6 mice with VML wounds.

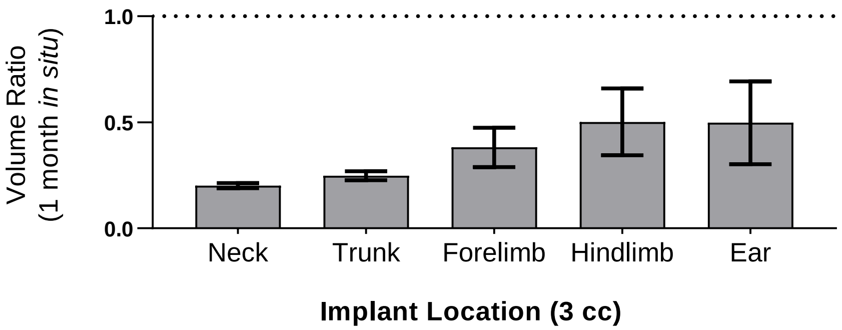

Supplementary Figure 5. Volume retention of pAAT implants at different anatomical sites in swine.

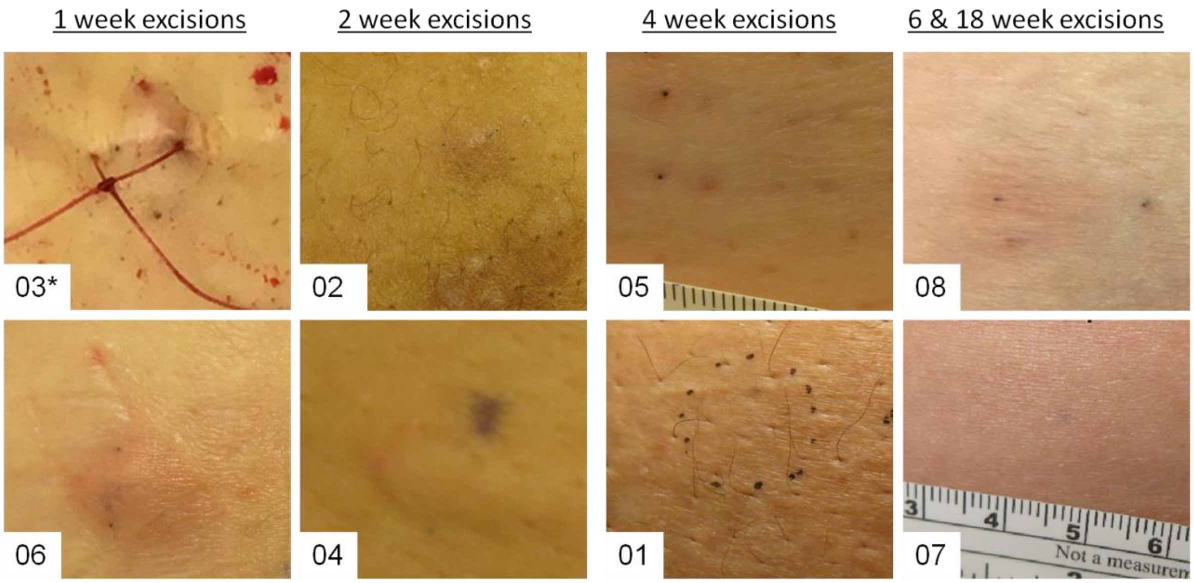

Supplementary Figure 6. Injection site photos at time of AAT implant excision for clinical subjects.

*Excision visit photo for Subject 03 was taken after their surgery.

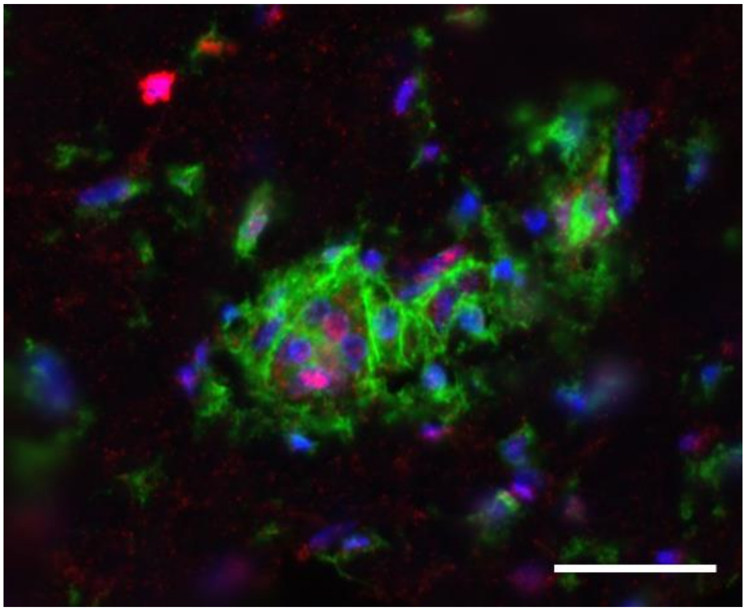

Supplementary Figure 7. Clusters of $CD4^+$ (green) and $CD8^+$ (red) T cells within AAT implants in human subject resemble small tertiary lymphoid structures (scale bar represents 50 µm, blue represents DAPI).

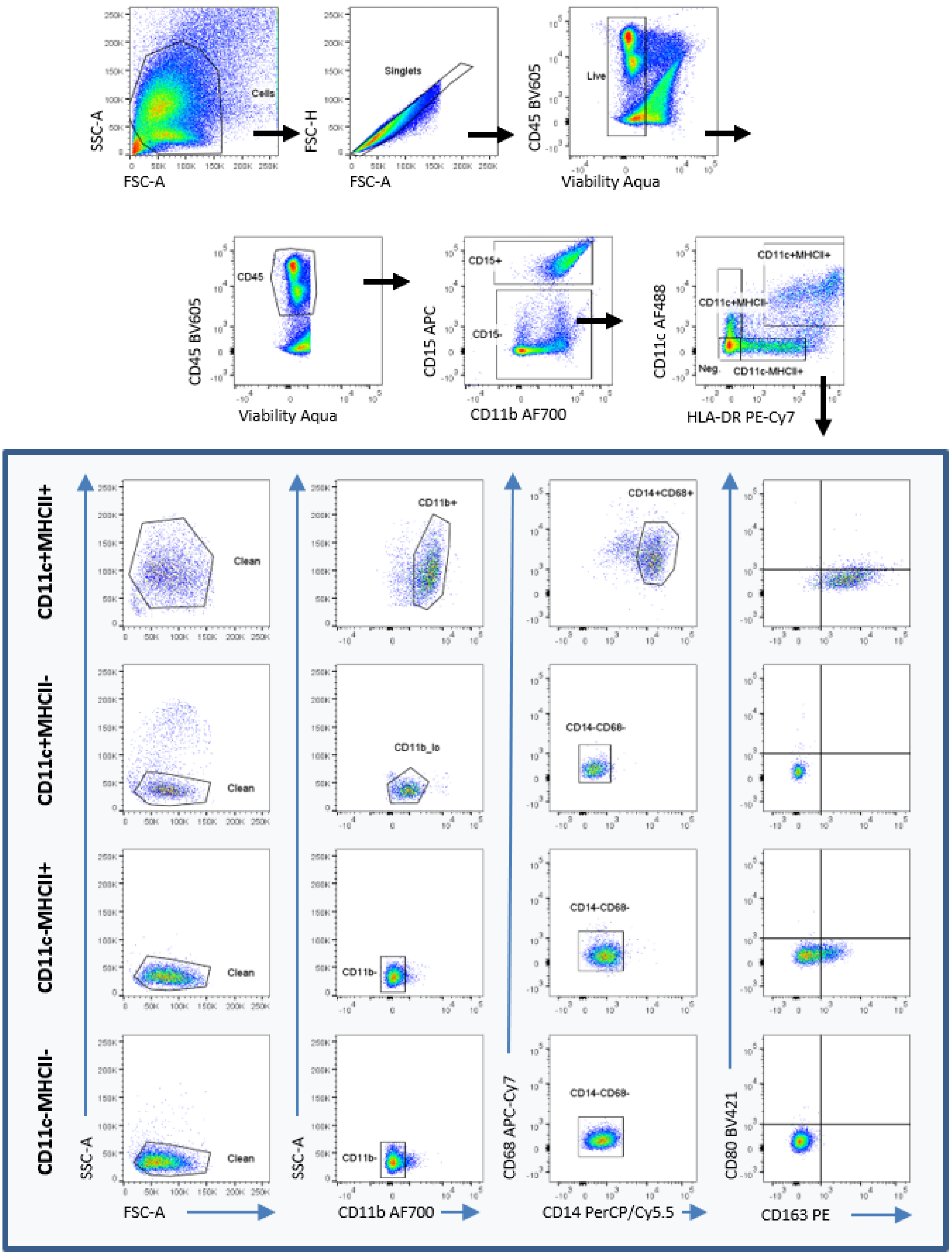

Supplementary Figure 8. Gating schema for human flow cytometry experiments assessing polarization markers in myeloid populations.

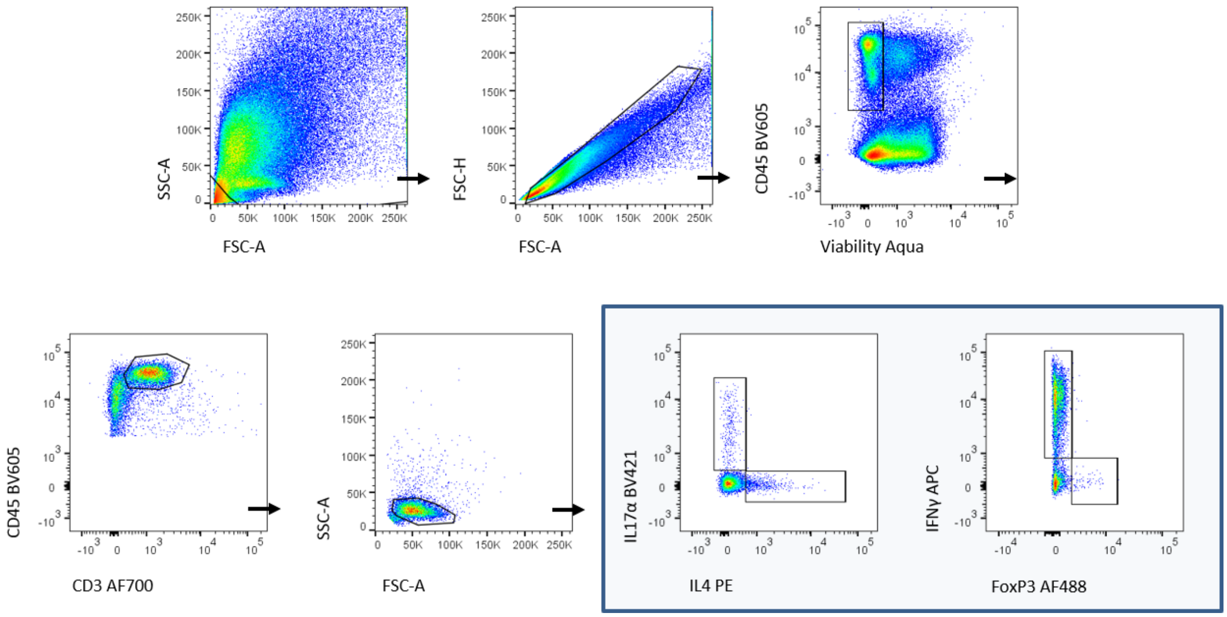

Supplementary Figure 9. Gating schema for human flow cytometry experiments assessing cytokine production in lymphoid populations.

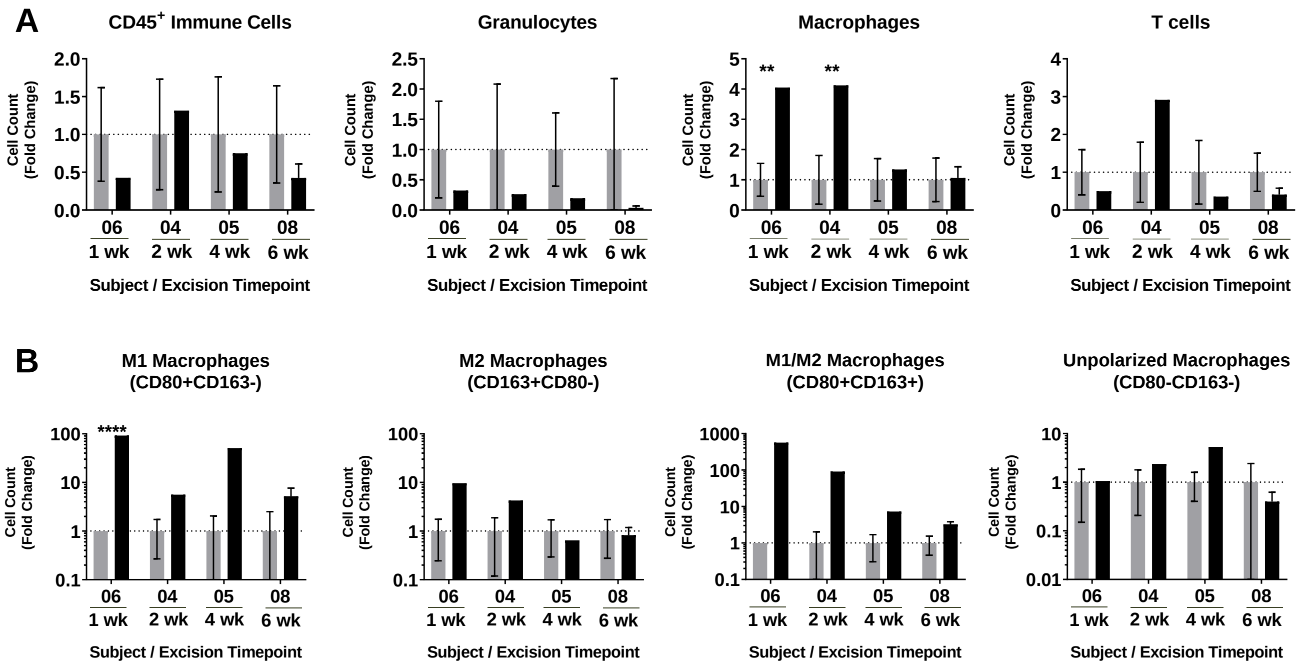

Supplementary Figure 10. Cell counts in human AAT implants (black bars) normalized to matched adipose tissue (grey bars) in four clinical subjects. (A) Relative quantification of total cell populations ($CD45^+$) and immune subsets, including granulocytes, macrophages, and T cells. (B) Numbers of macrophages with differential expression of polarization markers CD163 and CD80.

SUPPLEMENTARY TABLES

Supplementary Table 1. Proteomic characterization of AAT and ACD by LC-MS/MS.

CATEGORY	PROTEIN	Identifying peptides			
		ACD 1	ACD 2	AAT 1	AAT 2
Proteoglycan	Asporin	0	0	4	4
Proteoglycan	Heparin sulfate proteoglycan 2	0	0	2	4
Proteoglycan	Biglycan	1	3	7	10
ECM Glycoprotein	Cartilage intermediate layer protein	0	0	1	2
Collagen	Collagen, type I, alpha 1	29	36	17	21
Collagen	Collagen, type I, alpha 2	21	16	8	9
Collagen	Collagen, type III, alpha 1	5	9	3	3
Collagen	Collagen, type VI, alpha 1	6	6	9	14
Collagen	Collagen, type VI, alpha 2	3	8	7	9
Collagen	Collagen, type VI, alpha 3	19	28	36	66
Collagen	Collagen, type XIV, alpha 1	0	0	0	3
Proteoglycan	Decorin	8	9	6	5
ECM Glycoprotein	Dermatopontin	0	0	2	2
ECM Glycoprotein	Fibrillin 1	0	0	5	0
ECM Glycoprotein	Laminin, alpha 4	0	0	1	2
ECM Glycoprotein	Laminin, beta 2 (laminin S)	0	0	2	2
Proteoglycan	Lumican	0	2	5	12
Proteoglycan	Osteoglycin	7	6	10	13
ECM Glycoprotein	Periostin (Osteoblast specific factor)	1	2	0	0
Proteoglycan	Proline/arginine-rich end leucine-rich repeat protein	3	4	3	6
ECM-affiliated	Annexin A1	0	0	4	4
ECM-affiliated	Annexin A2	0	0	6	6
ECM-affiliated	Annexin A5	0	0	1	5
ECM-affiliated	Annexin A6	0	0	3	3
ECM-affiliated	Coagulation factor XIII, A1 polypeptide	0	0	0	2

Probability Legend

Over 95%
80% - 94%
50% - 79%

Supplementary Table 2. Statistical significance between each pair of experimental groups in Figures 4B and 4C

<u>Figure 4B - Flow cytometry plots</u>

M2 ($CD206^+CD86^-$ %)

	Uninj.	Saline	Syn-M	Allo-M	Xeno-P
Saline	ns				
Syn-M	****	***			
Allo-M	***	**	ns		
Xeno-P	****	****	****	****	
Xeno- H	****	****	****	****	ns

M1 ($CD206^-CD86^+$ %)

	Uninj.	Saline	Syn-M	Allo-M	Xeno-P
Saline	****				
Syn-M	**	****			
Allo-M	***	*	ns		
Xeno-P	ns	****	**	***	
Xeno- H	ns	****	*	**	ns

DP ($CD206^+CD86^+$ %)

	Uninj.	Saline	Syn-M	Allo-M	Xeno-P
Saline	****				
Syn-M	****	ns			
Allo-M	****	ns	ns		
Xeno-P	****	****	****	****	
Xeno- H	****	****	****	****	ns

M2 / M1 Ratio

	Uninj.	Saline	Syn-M	Allo-M	Xeno-P
Saline	ns				
Syn-M	****	***			
Allo-M	***	**	ns		
Xeno-P	****	****	****	****	
Xeno- H	****	****	****	****	ns

<u>Figure 4C – Gene Expression</u>

Il4

	Uninj.	Saline	Syn-M	Allo-M	Xeno-P
Saline	ns				
Syn-M	ns	ns			
Allo-M	ns	ns	ns		
Xeno-P	****	****	****	****	
Xeno- H	****	****	****	****	ns

Ifng

	Uninj.	Saline	Syn-M	Allo-M	Xeno-P
Saline	ns				
Syn-M	ns	ns			
Allo-M	ns	ns	ns		
Xeno-P	*	ns	ns	ns	
Xeno- H	****	**	*	***	ns

Arg1

	Uninj.	Saline	Syn-M	Allo-M	Xeno-P
Saline	ns				
Syn-M	ns	ns			
Allo-M	ns	ns	ns		
Xeno-P	****	****	****	****	
Xeno- H	****	****	****	****	*

Nos2

	Uninj.	Saline	Syn-M	Allo-M	Xeno-P
Saline	ns				
Syn-M	ns	ns			
Allo-M	ns	ns	ns		
Xeno-P	ns	*	ns	ns	
Xeno- H	**	***	**	*	ns

Supplementary Table 3. Injection site locations and discrete implant volumes in safety assessment of pig-adipose derived AAT in Yorkshire cross pigs.

Implant Number	Location	Volume (cc)
1	Right Trunk/flank	20
2	Left Trunk/flank	10
3	Left Ear	3
4	Left Cervical/neck, ventral	3
5	Left Trunk/flank	3
6	Left Medial forelimb	3
7	Left Medial hindlimb	3
8	Right Medial hindlimb	3
Total Dose / Animal:		48 cc

Supplementary Table 4. Pathologist scoring of pig AAT implants and surrounding tissue in swine study (Pig #1).

Study Title	Pathology Scoring: Swine Allogeneic AAT							
Time Point	1 Month							
Animal Number	1							
Implant Number	1	2	3	4	5	6	7	8
Implant Material	Pig AAT	Pig AAT	Pig AAT	Pig AAT	Pig AAT	Pig AAT	Pig AAT	Pig AAT
Location	L Ear	L Cervical / neck, ventral	L Trunk/ flank	L Trunk/ flank	L Medial forelimb	L Medial hindlimb	R Trunk/ flank	R. Medial hindlimb
TISSUE								
Cell Type/Response								
Polymorphonuclear cells	0	0	0	0	0	0	0	0
Lymphocytes	1	1	1	2	1	1	2	1
Plasma Cells	0	0	0	0	0	0	0	0
Macrophages	0	1	1	1	1	1	1	1
Giant Cells	0	0	0	0	0	0	0	0
Edema Fluid Accumulation	0	0	0	0	0	0	0	0
Necrosis	0	0	1M	1M	0	0	2M	0
Response								
Congestion	0	0	0	0	0	0	0	0
Mineralization	0	0	0	0	0	0	1M	0
Fibroplasia	0	0	0	0	0	0	0	0
Fibrosis	0	0	0	0	0	0	1M	0
IMPLANT								
Cell Type/Response								
Polymorphonuclear cells	0	0	0	0	0	0	0	0
Lymphocytes	2	2	2	2	3	2	3	3
Plasma Cells	1	1	1	1	1	1	1	1
Macrophages	1	1	1	2	2	1	1	1
Giant Cells	1	1	1	1	1	1	1	1
Edema Fluid Accumulation	0	0	0	0	0	0	0	0
Nonviable acellular matrix	0	0	0	2	1	0	3	0
Response								
Congestion	0	0	0	0	0	0	0	0
Mineralization	0	0	0	0	0	0	0	0
Fibroplasia	0	0	0	2	0	0	2	0
Fibrosis	4	4	4	4	4	4	3	4
Area(s) of dropout	P	NP	NP	P	P	NP	P	NP

Key: P = Present, NP = Not Present, 0 = None, 1 = Minimal, 2 = Mild, 3 = Moderate, 4 = Severe, R = Rare, M = associated with skeletal muscle

Supplementary Table 5. Pathologist scoring of pig AAT implants and surrounding tissue in swine study (Pig #2).

Study Title	Pathology Scoring: Swine Allogeneic AAT							
Time Point	1 Month							
Animal Number	2							
Implant Number	1	2	3	4	5	6	7	8
Implant Material	Pig AAT	Pig AAT	Pig AAT	Pig AAT	Pig AAT	Pig AAT	Pig AAT	Pig AAT
Location	L Ear	L Cervical / neck, ventral	L Trunk/ flank	L Trunk/ flank	L Medial forelimb	L Medial hindlimb	R Trunk/ flank	R. Medial hindlimb
TISSUE								
Cell Type/Response								
Polymorphonuclear cells	0	0	0	0	0	0	TM	0
Lymphocytes	2	2	2	1	2	2	TM	1
Plasma Cells	0	0	1	0	0	0	TM	0
Macrophages	1	1	1	1	1	1	TM	1
Giant Cells	1	0	0	0	0	0	TM	0
Edema Fluid Accumulation	0	0	0	0	0	0	TM	0
Necrosis	R-M	0	R-M	0	0	0	TM	0
Response								
Congestion	0	0	0	0	0	0	TM	0
Mineralization	0	0	0	0	0	0	TM	0
Fibroplasia	0	0	0	0	0	0	TM	0
Fibrosis	1	0	1	0	0	0	TM	0
IMPLANT								
Cell Type/Response								
Polymorphonuclear cells	0	0	0	0	0	0	TM	0
Lymphocytes	2	2	2	3	2	2	TM	2
Plasma Cells	1	1	1	1	1	1	TM	1
Macrophages	1	1	1	1	1	1	TM	1
Giant Cells	1	1	1	1	1	1	TM	1
Edema Fluid Accumulation	0	0	0	0	0	0	TM	0
Nonviable acellular matrix	1	0	0	2	0	1	TM	0
Response								
Congestion	0	0	0	0	0	0	TM	0
Mineralization	0	0	0	0	0	0	TM	0
Fibroplasia	0	0	0	0	0	0	TM	0
Fibrosis	4	4	4	4	4	4	TM	4
Area(s) of dropout	P	NP	NP	P	NP	P	TM	NP

Key: P = Present, NP = Not Present, 0 = None, 1 = Minimal, 2 = Mild, 3 = Moderate, 4 = Severe, R = Rare, M = associated with skeletal muscle

Supplementary Table 6. Pathologist scoring of pig AAT implants and surrounding tissue in swine study (Pig #3).

Study Title	Pathology Scoring: Swine Allogeneic AAT							
Time Point	1 Month							
Animal Number	3							
Implant Number	1	2	3	4	5	6	7	8
Implant Material	Pig AAT	Pig AAT	Pig AAT	Pig AAT	Pig AAT	Pig AAT	Pig AAT	Pig AAT
Location	L Ear	L Cervical / neck, ventral	L Trunk/ flank	L Trunk/ flank	L Medial forelimb	L Medial hindlimb	R Trunk/ flank	R. Medial hindlimb
TISSUE								
Cell Type/Response								
Polymorphonuclear cells	0	0	0	0	0	0	1	0
Lymphocytes	1	1	1	2	1	1	1	1
Plasma Cells	0	0	0	1	0	0	0	0
Macrophages	1	1	1	1	1	1	1	1
Giant Cells	0	0	0	R	0	0	0	0
Edema Fluid Accumulation	0	0	0	0	0	0	0	0
Necrosis	0	0	1M	1M	0	0	0	0
Response								
Congestion	0	0	0	0	0	0	0	0
Mineralization	0	0	0	0	0	0	0	0
Fibroplasia	0	0	0	0	0	0	0	0
Fibrosis	0	0	1	1	0	0	1M	0
IMPLANT								
Cell Type/Response								
Polymorphonuclear cells	0	0	0	0	1	1	1	0
Lymphocytes	1	2	1	2	3	2	2	2
Plasma Cells	0	1	0	1	1	1	1	1
Macrophages	1	2	1	1	2	1	2	1
Giant Cells	1	1	1	1	1	1	1	1
Edema Fluid Accumulation	0	0	0	0	0	0	0	0
Nonviable acellular matrix	1	0	0	2	1	1	3	0
Response								
Congestion	0	0	0	0	0	0	0	0
Mineralization	0	0	0	0	0	0	0	0
Fibroplasia	0	0	0	0	0	0	4	0
Fibrosis	4	4	4	4	4	4	4	4
Area(s) of dropout	P	NP	NP	NP	P	P	P	NP

Key: P = Present, NP = Not Present, 0 = None, 1 = Minimal, 2 = Mild, 3 = Moderate, 4 = Severe, R = Rare, M = associated with skeletal muscle

Supplementary Table 7. Summary of anticipated AEs noted by study visit in Phase 1 clinical testing.

Excision Time Point	1 week		2 weeks		4 weeks		6 wk	18 wk	Total
Subject ID	03	06	02	04	01	05	08	07	
Pain/Tenderness					Inj		1wk	1wk	3/8 (37.5%)
Erythema		Ex	Ex	1wk			1wk Ex		4/8 (50%)
Bruising		Ex				1wk	2wk	1wk	4/8 (50%)
Hyperpigmentation							Ex		1/8 (12.5%)
Textural change[1]						1wk 2wk	1wk 4wk Ex	1wk 2wk 4wk	3/8 (37.5%)
Other (define)	Ex Implant indurated			Ex Implants indurated		Ex Implant raised			3/8 (37.5%)

[1] Textural change was described as implant being "raised", "indurated", or "palpable"

Study Visit Key: Inj= Injection visit, 1wk= 1-week post-injection visit, 2wk= 2-week post-injection visit, 4wk= 4-week post-injection visit, Ex= Excision visit

Supplementary Table 8. Panel reactive antibody screening for Phase 1 subjects.

Subject ID	Excision Time point	BASELINE TESTING<br>IgG HLA Positive	POST-INJECTION TESTING<br>Increase in IgG HLA antibodies at:<br>*4 weeks*	*12 weeks*
03	1 week	Yes	No	No
06	1 week	No	No	Yes; lowest antibody detection level CPRA $_{Low}$ = 44
02	2 weeks	Participant withdrew prior to follow-up		
04	2 weeks	Yes	No	No
05	4 weeks	No	No	No
01	4 weeks	No	No	No
08	6 weeks	No	No	No
07	18 weeks	Yes	No	No

Supplementary Table 9. Human qRT-PCR Primers

Gene	Forward Sequence 5' – 3'	Reverse Sequence 5' – 3'
PPARG	AGGAGAAGCTGTTGGCGGAGA	TGCTTTGGTCAGCGGGAAGG
LPL	GTCAGAGCCAAAAGAAGCAGCAA	GGGTTTCACTCTCAGTCCCAGAA
LEP	TGACACCAAAACCCTCATCAAGACAA	GGAGCCCAGGAATGAAGTCCAA
CEBPA	TCACCGCTCCAATGCCTACTG	CCTGCTCCCCTCCTTCTCTCAT
FABP4	ACAGGAAAGTCAAGAGCACCATAACC	TGACGCATTCCACCACCAGTTT
B-ACTIN	GGCACCCAGCACAATGAA	GCTAACAGTCCGCCTAGAAGC

Supplementary Table 10. Murine qRT-PCR Primers

Gene	Forward Sequence 5' – 3'	Reverse Sequence 5' – 3'
Il4	GGTCACAGGAGAAGGGACGC	AGCACCTTGGAAGCCCTACA
Ifng	GTCAGAGCCAAAAGAAGCAGCAA	TGTCACCATCCTTTTGCCAGT
Arg1	ACAAGACAGGGCTCCTTTCAG	TAAAGCCACTGCCGTGTTCA
Nos2	CTTGGTGAAGGGACTGAGCTG	GTTCTCCGTTCTCTTGCAGTTG
B2m	CACTGAATTCACCCCCACTGA	TCTCGATCCCAGTAGACGGT
