# Supplementary material for: An immunologically active, adipose-derived extracellular matrix biomaterial for soft tissue reconstruction: concept to clinical trial
Source: NPJ Regen Med. 2022 Jan 14;7:6. doi: 10.1038/s41536-021-00197-1 (PMC8760240; doi:10.1038/s41536-021-00197-1)
Supplement: Supplementary file 2 — Reporting Summary [file 41536_2021_197_MOESM2_ESM.pdf]

nature research

Corresponding author(s): Jennifer Elisseeff
Last updated by author(s): Jun 7, 2021

Reporting Summary

Nature Research wishes to improve the reproducibility of the work that we publish. This form provides structure for consistency and transparency in reporting. For further information on Nature Research policies, see our Editorial Policies and the Editorial Policy Checklist.

Statistics

For all statistical analyses, confirm that the following items are present in the figure legend, table legend, main text, or Methods section.

n/a	Confirmed	
☐	☒	The exact sample size (*n*) for each experimental group/condition, given as a discrete number and unit of measurement
☐	☒	A statement on whether measurements were taken from distinct samples or whether the same sample was measured repeatedly
☐	☒	The statistical test(s) used AND whether they are one- or two-sided<br>*Only common tests should be described solely by name; describe more complex techniques in the Methods section.*
☐	☒	A description of all covariates tested
☐	☒	A description of any assumptions or corrections, such as tests of normality and adjustment for multiple comparisons
☐	☒	A full description of the statistical parameters including central tendency (e.g. means) or other basic estimates (e.g. regression coefficient) AND variation (e.g. standard deviation) or associated estimates of uncertainty (e.g. confidence intervals)
☒	☐	For null hypothesis testing, the test statistic (e.g. *F*, *t*, *r*) with confidence intervals, effect sizes, degrees of freedom and *P* value noted<br>*Give P values as exact values whenever suitable.*
☒	☐	For Bayesian analysis, information on the choice of priors and Markov chain Monte Carlo settings
☒	☐	For hierarchical and complex designs, identification of the appropriate level for tests and full reporting of outcomes
☒	☐	Estimates of effect sizes (e.g. Cohen's *d*, Pearson's *r*), indicating how they were calculated

Our web collection on statistics for biologists contains articles on many of the points above.

Software and code

Policy information about availability of computer code

Data collection	FEI Quanta 200 SEM, Mascot Daemon, FACSDIVA, StepONE Plus
Data analysis	Scaffold 3, Proteome Discoverer, ImageJ, FlowJo, GraphPad Prism, Microsoft Excel

For manuscripts utilizing custom algorithms or software that are central to the research but not yet described in published literature, software must be made available to editors and reviewers. We strongly encourage code deposition in a community repository (e.g. GitHub). See the Nature Research guidelines for submitting code & software for further information.

Data

Policy information about availability of data

All manuscripts must include a data availability statement. This statement should provide the following information, where applicable:
- Accession codes, unique identifiers, or web links for publicly available datasets
- A list of figures that have associated raw data
- A description of any restrictions on data availability

All data associated with this study are present in the paper or the Supplementary Materials. All primary data and analyses related to this manuscript are available upon request.

Field-specific reporting

Please select the one below that is the best fit for your research. If you are not sure, read the appropriate sections before making your selection.

☒ Life sciences ☐ Behavioural & social sciences ☐ Ecological, evolutionary & environmental sciences

For a reference copy of the document with all sections, see nature.com/documents/nr-reporting-summary-flat.pdf

Life sciences study design

All studies must disclose on these points even when the disclosure is negative.

Sample size	Sample sizes were chosen based on prior experience with the animal models for tissue engineering applications. Sample size in the FIH study was determined to investigate escalating doses and times in situ for AAT implants and related safety measurements, but was not designed to reach statistically significant conclusions on exploratory outcomes.
Data exclusions	No data were excluded from these analyses.
Replication	Assay validation experiments were performed in advance where ever possible to ensure robustness and reproducibility of experimental methods. When additional treatment groups were investigated, control groups were repeated and compared to previous experiments to verify that run-to-run variability was not significant. These attempts to validate reproducibility were successful in all data presented here.
Randomization	In animal studies, cages were randomized to different treatment groups. In the FIH study, all volunteers received the same study intervention and dosing levels/duration in situ were determined by order of enrollment and other factors relating to the schedule of the participants, therefore randomization was not possible.
Blinding	Blinding to group allocation was not possible in animal or human studies. In animal studies, investigators could easily visually distinguish between treatment materials (AAT vs. lipoaspirate, AAT vs. saline). All human subjects received the same study intervention in the Phase I study.

Reporting for specific materials, systems and methods

We require information from authors about some types of materials, experimental systems and methods used in many studies. Here, indicate whether each material, system or method listed is relevant to your study. If you are not sure if a list item applies to your research, read the appropriate section before selecting a response.

Materials & experimental systems

n/a	Involved in the study	
☐	☒	Antibodies
☒	☐	Eukaryotic cell lines
☒	☐	Palaeontology and archaeology
☐	☒	Animals and other organisms
☐	☒	Human research participants
☐	☒	Clinical data
☒	☐	Dual use research of concern

Methods

n/a	Involved in the study	
☒	☐	ChIP-seq
☐	☒	Flow cytometry
☒	☐	MRI-based neuroimaging

Antibodies

Antibodies used	In 4get studies, isolated cells were stained on ice with LIVE/DEAD Fixable Aqua viability dye (Thermo Fisher) followed by a surface marker cocktail: CD45 Brilliant Violet 605 (Clone 30-F11), CD11b Alexa Fluor 700 (Clone M1/70), CD3 BB700 (Clone 145-2C11, BD Biosciences), Ly-6c Brilliant Violet 510 (Clone HK1.4), Ly-6g Pacific Blue (Clone 1A8), F4/80 PE-Cy7 (Clone BM8), MHCII I-A/I-E PE-594 (Clone M5/114.15.3), Siglec-F Brilliant Violet 711 (Clone E50-2440, BD Biosciences), CD4 APC (Clone GK1.5), and CD8 PE (Clone 53-6.7). All antibodies were obtained from BioLegend.<br><br>In C57BL/6 studies, isolated cells were stained using the following cocktail of surface markers: CD45 Brilliant Violet 605 (Clone 30-F11), CD11b Alexa Fluor 700 (Clone M1/70), CD11c PerCP/Cy5.5 (Clone N418), CD3 PE-Cy5 (Clone 145-2C11), Ly-6c Brilliant Violet 510 (Clone HK1.4), Ly-6g Pacific Blue (Clone 1A8), F4/80 PE-Cy7 (Clone BM8), MHCII I-A/I-E Alexa Fluor 488 (Clone M5/114), Siglec-F PE-594 (Clone E50-2440, BD Biosciences), CD206 PE (Clone C068C2), and CD86 APC (Clone GL-1). All antibodies were obtained from BioLegend.<br><br>In human flow cytometry experiments, myeloid panel surface markers included: CD45 Brilliant Violet 605 (Clone HI30), CD11b Alexa Fluor 700 (Clone M1/70), CD11c Alexa Fluor 488 (Clone 3.9), CD14 PerCP/Cy5.5 (Clone HCD14), CD15 APC (Clone W6D3), HLA-DR/DP/DQ PE-Cy7 (Clone Tu39), CD80 Brilliant Violet 421 (Clone 2D10), and CD163 PE (Clone GHI/61). Lymphoid markers were stained in a second panel of surface and intracellular markers, including: CD45 Brilliant Violet 605 (Clone HI30), CD3 Alexa Fluor 700 (Clone SK7),

	IL4 PE (Clone 8D4-8), IFNγ APC (Clone 4S.B3), IL17α Brilliant Violet 421 (Clone BL168), and FoxP3 Alexa Fluor 488 (Clone 150D). All antibodies were obtained from BioLegend.
Validation	All antibodies for flow cytometry were be purchased from eBioscience/Thermofisher, Biolegend, or BD Biosciences and were titrated within the Flow Cytometry Technology Development Center (FCTC) of the Bloomberg Kimmel Institute at Johns Hopkins to determine the optimal staining concentration. Isotype controls were used whenever possible. Staining was performed on control tissues to test the specificity and quality of the antibodies. Antibodies were discarded at expiration to maintain optimal quality and reproducibility.

Animals and other organisms

Policy information about studies involving animals; ARRIVE guidelines recommended for reporting animal research

Laboratory animals	Athymic nude mice, female, six weeks old<br>Wild-type C57BL/b mice, female, six to eight weeks old<br>4get mice, female, six to eight weeks old<br>CD-1 mice, male, retired breeders > 18 weeks old<br>Yorkshire cross pigs, female, 2.5 months old
Wild animals	Study did not involve wild animals.
Field-collected samples	Study did not involve field-collected samples.
Ethics oversight	All animal procedures were approved by Johns Hopkins Institutional Care and Use Committee (IACUC). All patient procedures were approved by the Johns Hopkins Institutional Review Board.

Note that full information on the approval of the study protocol must also be provided in the manuscript.

Human research participants

Policy information about studies involving human research participants

Population characteristics	Eight human subjects participated in the study. Seven out of 8 subjects were female. Ages ranged from 27-65 years with a mean age of 48 years. After receiving AAT injections, implants were recovered following an elective surgical procedure to remove unwanted tissue. Five subjects underwent a panniculectomy and three subjects underwent an abdominoplasty.
Recruitment	Patients were recruited by protocol physicians through the plastic surgery clinics at Johns Hopkins Medicine (ie: Johns Hopkins Hospital, Johns Hopkins Bayview Medical Center, Johns Hopkins White Marsh Medical Center). Although no bias towards gender, race, or ethnicity was outlined in the pilot study protocol, self-selection bias was likely present as all study participants were Caucasian and 7 of 8 were female. The study design required individuals undergoing elective surgical procedures so bias towards a specific gender and socio-economic factors are likely represented in the study population. The impact of this potential bias in the study population on clinical response data or immunological findings is unknown. Gender-based or racially-based differences may be present and not identified in this study.
Ethics oversight	A Phase I clinical study was conducted at the Johns Hopkins University School of Medicine (Baltimore, MD) with approval by the Johns Hopkins University IRB.

Note that full information on the approval of the study protocol must also be provided in the manuscript.

Clinical data

Policy information about clinical studies
All manuscripts should comply with the ICMJE guidelines for publication of clinical research and a completed CONSORT checklist must be included with all submissions.

Clinical trial registration	NCT02817984
Study protocol	We have not made the full protocol public. We have not done so partly out of proprietary nature of project/AAT development. Main data collection info, etc, is available on ClinicalTrials.gov.
Data collection	Subjects were recruited by protocol physicians and team members through the plastic surgery clinics at Johns Hopkins Medicine (ie: Johns Hopkins Hospital, Johns Hopkins Bayview Medical Center, Johns Hopkins White Marsh Medical Center). Study visits were also performed at these locations. Study recruitment took place between 03/01/2016 and 10/14/2016. Data was collected between 04/15/2016 and 04/30/2017.
Outcomes	The primary outcome measured was safety of acellular adipose tissue (AAT) injections as determined by the incidence of adverse (up to 12 weeks post-injection). Secondary outcome measures assessed the histopathology of explanted implants via hematoxylin and eosin (H&E) staining and flow cytometry as well as tolerability of AAT injections per the participant-reported experience (up to 12 weeks post-injection).

Flow Cytometry

Plots

Confirm that:

☒ The axis labels state the marker and fluorochrome used (e.g. CD4-FITC).

☒ The axis scales are clearly visible. Include numbers along axes only for bottom left plot of group (a 'group' is an analysis of identical markers).

☒ All plots are contour plots with outliers or pseudocolor plots.

☒ A numerical value for number of cells or percentage (with statistics) is provided.

Methodology

Sample preparation	For studies in mice, harvested quadriceps tissue and any associated biomaterial were pooled for each individual mouse, then samples were finely diced in 1X DPBS on ice, digested for 45 minutes at 37°C in an enzyme solution consisting of 1.67 Wunsch U/mL Liberase TL (Sigma-Aldrich) and 0.2 mg/mL DNAse I (Roche) in RPMI 1640, and filtered sequentially through 100 µm and 70 µm cell strainers prior to staining. For our human study, analysis of immune cell recruitment and cytokine expression was performed on dissociated AAT implants and normal adjacent tissue samples using separate panels for myeloid and lymphoid markers. Specimens were finely diced in 1X DPBS on ice, digested for 45 minutes at 37°C in an enzyme solution consisting of 1.67 Wunsch U/mL Liberase TL (Sigma-Aldrich) and 0.2 mg/mL DNAse I (Roche) in RPMI 1640, and filtered sequentially through 100 µm and 70 µm cell strainers. For intracellular staining, cells were stimulated for 4 hours at 37°C in RPMI 1640 media (Gibco) with Cell Stimulation Cocktail Plus Protein Transport Inhibitors (eBioscience) prior to staining.
Instrument	BD LSRII flow cytometer
Software	Data was collected using BD FACSDIVA software and analyzed using FlowJo software.
Cell population abundance	No cell sorting or purification steps were performed. All cells isolated from disassociated tissue samples using the above methods were stained and analyzed by flow cytometry.
Gating strategy	A preliminary FSC-A/SSC-A gate was used to select total leukocytes and exclude small debris. A secondary gate was used to capture single cells (FSC-W/FSC-H). Single cells were then gated on viability and other fluorescent lineage or phenotypic markers. Gating of fluorescent markers was determined based on fluorescence minus one (FMO) plus isotype controls.

☒ Tick this box to confirm that a figure exemplifying the gating strategy is provided in the Supplementary Information.
